# Supplementary material for: Causal Relationship between Aging and Anorexia Nervosa: A White-Matter-Microstructure-Mediated Mendelian Randomization Analysis
Source: Biomedicines. 2024 Aug 16;12(8):1874. doi: 10.3390/biomedicines12081874 (PMC11351342; doi:10.3390/biomedicines12081874)
Supplement: Supplementary file 1 [file biomedicines-12-01874-s001.zip › Supplementary Figure/Figure S8/Leaveoneout plot of MR analysis for TL to ubm-b-1841.pdf]

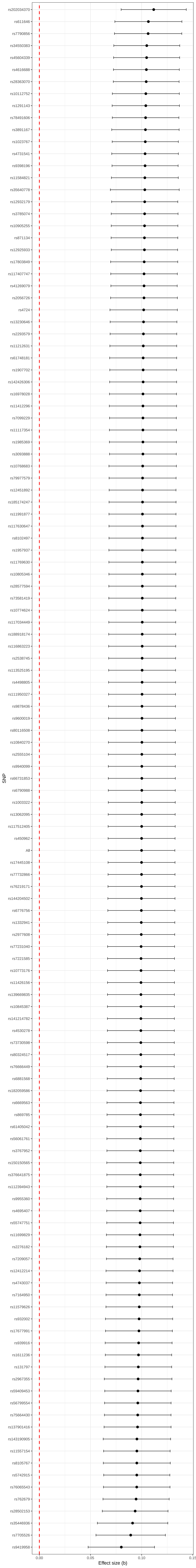
SNP
Effect size (b)
0.00
0.05
0.10
0.15
rs202034370
rs611646
rs7790856
rs34550383
rs45604339
rs4616688
rs28363070
rs10112752
rs1291143
rs78491606
rs3891167
rs1023767
rs4731541
rs9398196
rs11584821
rs35640778
rs12932179
rs3785074
rs10905255
rs871134
rs12925933
rs17803849
rs117407747
rs41269079
rs2056726
rs4724
rs13230646
rs2293579
rs11212631
rs61748181
rs1907702
rs142426306
rs16978028
rs11412296
rs7099229
rs11117354
rs1985369
rs3093888
rs10768683
rs79977579
rs12451892
rs185174247
rs11991877
rs117630647
rs8102497
rs1957937
rs11769630
rs10805346
rs28577594
rs73581419
rs10774624
rs117034449
rs188918174
rs116863223
rs2538745
rs113525195
rs4498805
rs111950327
rs9878436
rs9600619
rs80116508
rs10840270
rs2555104
rs9940099
rs66731853
rs6790988
rs1003322
rs13062095
rs117512405
rs450962
All
rs17445108
rs77732866
rs76219171
rs144204502
rs6776756
rs1332941
rs2977608
rs77231040
rs7221585
rs10773176
rs11426156
rs139669835
rs10845387
rs141214782
rs4530278
rs73730598
rs80324517
rs76666449
rs6881568
rs182059586
rs6669563
rs869785
rs61405042
rs56061761
rs3767952
rs150150565
rs376641875
rs112394943
rs9955360
rs4695407
rs55747751
rs11699829
rs2276182
rs7209057
rs12412214
rs4743037
rs7164950
rs11579626
rs932002
rs17677991
rs939916
rs1611236
rs131797
rs2967355
rs59409453
rs56799554
rs75664430
rs137901416
rs143190905
rs11557154
rs8105767
rs5742915
rs76065543
rs762679
rs28502153
rs35446936
rs7705526
rs9419958
